# Phytochemical Composition and Bioactivity of *Acanthus dioscoridis* var. *perringii*: An Integrated Analysis of Antioxidant Activity, Enzyme Inhibition, and Phenolic–Bioactivity Correlations

**DOI:** 10.3390/ph19030512

**Published:** 2026-03-20

**Authors:** Bedrettin Selvi

**Affiliations:** Department of Biology, Faculty of Arts and Sciences, Tokat Gaziosmanpaşa University, 60250 Tokat, Türkiye; bedrettin.selvi@gop.edu.tr; Tel.: +90-530-692-37-49

**Keywords:** *Acanthus dioscoridis* var. *perringii*, phenolic profile, LC-ESI-MS/MS, antioxidant activity, enzyme inhibition, bioactivity relationships

## Abstract

**Objectives:** Plant organs often allocate phenolic metabolites unevenly, resulting in organ-specific bioactivities. This study aimed to characterize the organ-specific phenolic profile of *Acanthus dioscoridis* var. *perringii* and determine how this chemical segregation is associated with antioxidant capacity and enzyme inhibitory activities. **Materials and Methods:** Organ-specific extracts (roots, stems, leaves, bracts, and flowers) were evaluated for total phenolic and flavonoid contents, targeted LC-MS analysis of individual phenolics, antioxidant activity by multiple assays, enzyme inhibition [acetylcholinesterase (AChE), butyrylcholinesterase (BChE), α-amylase, and α-glucosidase], and the relationships between phenolic composition and biological activities. Antioxidant performance was also assessed using the Relative Antioxidant Capacity Index (RACI). **Results and Discussion:** Roots showed the highest total phenolic content, whereas bracts had the highest total flavonoid level. Verbascoside was the dominant compound in all organs, with the highest levels in leaves, roots, and bracts. Roots exhibited the strongest reducing and radical-scavenging activities, while flowers showed the best metal-chelating capacity. Enzyme inhibition was organ-dependent and generally moderate, with stems showing the strongest cholinesterase inhibition, leaves the strongest α-amylase inhibition, and bracts together with roots the strongest α-glucosidase inhibition. Statistical analysis revealed close associations between phenolic richness, antioxidant responses, and cholinesterase inhibition. **Conclusions:** These findings demonstrate a clear organ-dependent distribution of phenolic compounds in *A. dioscoridis* var. *perringii*, reflected in distinct antioxidant and enzyme inhibitory profiles. Overall, the study provides a biochemical and bioactivity-based characterization of the species at the organ level.

## 1. Introduction

Medicinal and aromatic plants represent a major focus of research on the prevention and management of chronic diseases due to their wide variety of secondary metabolites. Within this diversity, phenolic compounds, in particular, are being extensively studied for their role in regulating biological processes such as oxidative stress and inflammation, and their protective potential against cardiovascular, neurodegenerative, and metabolic disorders is frequently emphasized [1,2]. Flavonoids, phenolic acids, and related derivatives are among the most important components of plant-derived therapeutics, largely because they can neutralize free radicals, chelate metal ions, and inhibit certain enzymes [3,4]. Therefore, detailed documentation of the phenolic profiles and associated biological activities of wild plants has become one of the main goals of natural product research.

*Acanthus* species (Acanthaceae) have long held a prominent position in pharmaceutical terms due to their widespread use in traditional medicine for anti-inflammatory, hepatoprotective, and dermatological purposes [5]. Phytochemical studies on the species indicate that the *Acanthus* genus is rich in phenolic compounds, flavonoids, triterpenoids, saponins, and alkaloids, and that these compounds have been reported to exhibit antioxidant, antimicrobial, and anticancer activities [5,6]. In this context, various *Acanthus* species, especially *A. ilicifolius*, have been shown to be among the most promising sources of biologically active natural products in recent years.

Studies on *Acanthus dioscoridis* L. are largely limited to var. *dioscoridis*. The antioxidant, antimicrobial, and cytotoxic effects of this variety, as well as its mineral and lipid composition, have been reported in detail [7,8]. Recent studies have demonstrated that leaf extracts exhibit significant wound-healing activity related to their high phenolic content, and that this effect has been associated with the regulation of inflammatory processes [9]. However, there are no comprehensive studies on the phenolic composition or biological activity of *A. dioscoridis* var. *perringii* (Siehe) E. Hossain, indicating a significant gap in the literature.

Phenolic composition may vary among plant species and also among different organs of the same species [10,11,12]. In this context, investigating the organ-specific phenolic profiles of *A. dioscoridis* var. *perringii* is relevant for documenting its chemical diversity. The present study was therefore designed as a phytochemical and bioactivity characterization of this taxon rather than as an ecological or physiological investigation.

The biological importance of phenolic compounds is not limited to oxidative stress regulation. These molecules have the potential to inhibit physiologically critical enzymes such as acetylcholinesterase (AChE), butyrylcholinesterase (BChE), α-amylase, α-glucosidase, and tyrosinase. Therefore, phenolic-rich extracts are considered as natural inhibitor sources in conditions such as neurodegenerative diseases, diabetes, and hyperpigmentation [13,14]. LC-MS-supported studies on various medicinal plants belonging to the flora of Turkey (*Achillea pseudoaleppica*, *Muscari armeniacum*, etc.) indicate that evaluation of the phenolic profile together with multiple antioxidant and enzyme inhibition analyses is a critical approach to identifying biologically potent species [15,16].

This study presents the first comprehensive phytochemical and biological activity investigation of *A. dioscoridis* var. *perringii* collected from the gypsum-rich steppe soils of central Anatolia. Specifically, the study addresses the following research question: whether different plant organs of *A. dioscoridis* var. *perringii* exhibit distinct phenolic compositions and associated antioxidant and enzyme inhibitory activities. The aims of the study were to determine the total phenolic and flavonoid contents and selected phenolic compounds in ethanolic extracts obtained from the flowers, leaves, stems, roots, and bracts of the plant by LC-ESI-MS/MS, to evaluate their in vitro antioxidant capacity using multiple antioxidant assays, to determine the inhibitory effects on AChE, BChE, α-amylase, α-glucosidase, and tyrosinase, and to elucidate the relationships between phenolic composition and biological activities by Relative Antioxidant Capacity Index (RACI)-based ranking and correlation analyses. Accordingly, this study is more appropriately framed as an organ-specific phytochemical and bioactivity profiling effort that integrates targeted phenolic analysis with multiple antioxidant and enzyme inhibition assays. In addition to providing the first detailed biochemical dataset for *A. dioscoridis* var. *perringii*, the work also contributes a chemotaxonomic perspective by documenting organ-dependent metabolite distribution patterns within the taxon. Because no direct validation at the compound or pathway level was performed, the findings should be regarded primarily as a basis for future targeted phytochemical and functional studies.

## 2. Results and Discussion

### 2.1. Chemical Composition

The organ-dependent differences were interpreted within the analytical scope of the present dataset.

**Figure 1:**
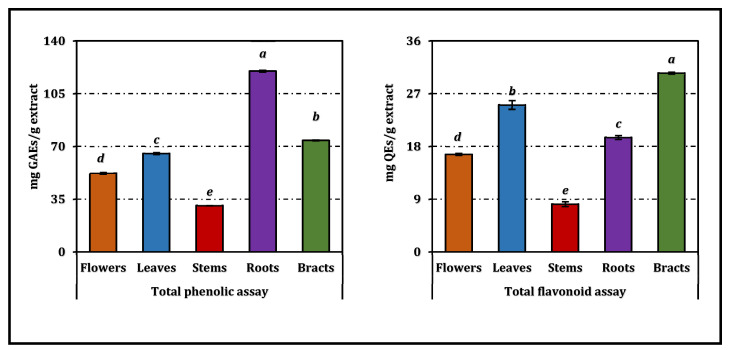
Total phenolic and flavonoid contents of *A. dioscoridis* var. *perringii* extracts. GAEs and REs: Gallic acid and rutin equivalents, respectively. Values indicated by the same superscripts (a–e) within the same column are not significantly different according to Tukey’s HSD test at the 5% significance level.

Total phenolic and flavonoid contents differed markedly among the organ-specific extracts of *A. dioscoridis* var. *perringii* (Figure 1). Roots exhibited by far the highest total phenolic content, reaching almost 120 mg GAEs/g extract and forming a statistically distinct group from all other organs. Bracts and leaves followed with intermediate TPC values, whereas flowers contained slightly lower amounts, and stems showed the poorest phenolic enrichment, in agreement with their separate significance groups. In contrast, total flavonoid content was maximized in bracts (around 30 mg REs/g extract), which again differed significantly from the remaining organs. Leaves and roots formed intermediate clusters in terms of flavonoids, while flowers and especially stems contained the lowest levels, consistent with their lower statistical categories (Figure 1). Overall, these data indicate a clear organ-dependent partitioning of both total phenolics and flavonoids, with below-ground tissues being particularly enriched in phenolics and bracts serving as the main flavonoid reservoir.

LC-MS-based quantification confirmed that this pattern is underpinned by substantial qualitative and quantitative variation in individual phenolics (Table 1). Verbascoside was the predominant constituent in all organs, with the highest levels occurring in the leaves, roots, and bracts, whereas the remaining organs contained comparatively lower amounts. Several hydroxybenzoic acids (3- and 4-hydroxybenzoic acids) and their derivatives were also abundant in aerial parts, with flowers generally showing the highest or comparable levels, followed by leaves and bracts, and much lower concentrations in the stems and roots. Flavone glycosides such as luteolin 7-glucoside, hyperoside, and apigenin 7-glucoside were principally concentrated in flowers and bracts, while leaves contained moderate amounts and the stems and roots remained comparatively poor. Aglycone forms such as luteolin and apigenin similarly peaked in flowers and bracts, with roots either containing trace amounts or lacking certain compounds (e.g., apigenin was not detected in roots). Phenolic acids including *p*-coumaric, ferulic, chlorogenic, rosmarinic, and sinapic acids were detected in all or most organs but with organ-specific maxima: flowers and leaves typically ranked among the richest sources, whereas stems and roots often displayed reduced levels. Several additional target phenolics were not detected in any of the analyzed extracts (Table 1). Collectively, Figure 1 and Table 1 demonstrate that *A. dioscoridis* var. *perringii* exhibits a highly differentiated phenolic profile across organs, dominated by verbascoside and a subset of hydroxybenzoic and flavone derivatives, highlighting clear organ-specific metabolic patterns rather than isolated compound-level variations.

The higher total phenolic content of the roots, despite the greater abundance of some individual compounds in the leaves and bracts, likely reflects the broader response of the Folin–Ciocalteu assay compared with the targeted LC-MS panel.

Taken together, these findings demonstrate a clear organ-specific phenolic distribution pattern and provide a useful chemotaxonomic reference within Acanthus.

The present investigation constitutes the first detailed account of the organ-specific phenolic architecture of *A. dioscoridis* var. *perringii*, providing previously unavailable comparative and chemotaxonomic insights for the genus. The pronounced heterogeneity observed among organs, particularly the enrichment of roots in total phenolics and the prominence of bracts as the major flavonoid reservoir, indicates a tightly regulated metabolic partitioning among tissues. These results indicate clear tissue-level differences in phenolic accumulation, although the present dataset does not allow for a physiological interpretation of the underlying causes.

Comparison with the limited phytochemical data available for *A. dioscoridis* var. *dioscoridis* highlights both shared metabolic motifs and notable divergences. Keskin [7] documented considerable variability in total phenolics and flavonoids among different solvent extracts of var. *dioscoridis*, suggesting that extraction polarity strongly influences the detectable phenolic spectrum. The much broader organ-level resolution provided here complements this solvent-based perspective by revealing that var. *perringii* exhibits similarly rich phenolic pools, yet distributes them in a more differentiated manner across tissues. Moreover, the present LC–MS/MS results demonstrate that verbascoside is a dominant constituent throughout the plant. This is noteworthy because earlier studies on var. *dioscoridis* did not profile verbascoside in detail, although the general abundance of caffeic acid derivatives reported in flowers [8] is consistent with the biochemical pathways leading to verbascoside formation.

The organ-dependent fluctuation of individual phenolic acids in var. *perringii*—particularly the prominence of hydroxybenzoic acid derivatives in flowers and leaves—also resonates with the high gallic and vanillic acid concentrations documented for the flowers of var. *dioscoridis* [8]. Yet, unlike the flower-centric distribution seen in that study, the present dataset points to a somewhat more balanced allocation across reproductive and vegetative organs in var. *perringii*. This suggests that phenolic biosynthesis may respond not only to developmental status, but also to genetic and microhabitat differences associated with taxonomic variation [17]. The high phenolic signatures reported for leaf extracts of *A. dioscoridis* in a recent in vivo study [18] further reinforce the genus-level trend that leaf tissues typically harbor substantial phenolic reservoirs—an observation echoed, though with different compound-level composition patterns, in the current work.

Perhaps equally informative are the compounds that remained undetected in var. *perringii*. The absence of catechins, epicatechin, certain cinnamic acid derivatives, and flavanones such as eriodictyol contrasts with the presence of related metabolites (e.g., naringenin, resveratrol) previously reported in var. *dioscoridis* [8]. These qualitative discrepancies may serve as chemotaxonomic markers distinguishing the two varieties, although broader population-level sampling is needed to evaluate the stability of such traits. The predominance of flavone glycosides and hydroxybenzoic acid derivatives in var. *perringii*, together with the widespread presence of verbascoside, points to a characteristic phenolic pattern in this taxon. However, the present data are not sufficient to support ecological or physiological interpretation of this pattern.

The current findings fill a gap in the phytochemical characterization of *A. dioscoridis* and provide a useful reference for future phytochemical, comparative, and taxonomic studies. Broader sampling and additional targeted analyses would be needed to determine the stability of the compositional patterns documented here.

### 2.2. Antioxidant Activity

Antioxidant responses differed markedly among the organs of *A. dioscoridis* var. *perringii* (Table 2). In the phosphomolybdenum assay, the root extract exhibited the highest total antioxidant capacity with an EC_50_ of 0.72 mg/mL, distinctly outperforming the leaves (0.94 mg/mL) and flowers (1.08 mg/mL), while the stems showed the weakest activity (1.34 mg/mL). A parallel pattern emerged in the cupric ion reducing antioxidant capacity (CUPRAC) assay, where the roots again displayed strong reducing power (0.35 mg/mL) compared with the bracts (0.52 mg/mL) and leaves (0.68 mg/mL), whereas stems retained the lowest performance (1.45 mg/mL). The FRAP assay further reinforced this trend: roots exhibited the most efficient ferric-reducing ability (0.21 mg/mL), followed by bracts (0.35 mg/mL) and leaves (0.40 mg/mL), while flowers (0.55 mg/mL) and stems (0.82 mg/mL) were less active.

Radical-scavenging assays also identified roots as the most potent fraction. In the DPPH test, the IC_50_ of the roots (0.77 mg/mL) was notably lower than those of the bracts (1.75 mg/mL) and leaves (2.18 mg/mL), whereas stems had the weakest activity (5.05 mg/mL). Similarly, in ABTS, roots yielded an IC_50_ of 0.71 mg/mL, in contrast to flowers (1.62 mg/mL) and stems (2.38 mg/mL). As expected, Trolox exhibited superior activity across these assays, confirming assay validity.

The ferrous ion chelating assay produced a different profile from the electron-transfer and radical-scavenging antioxidant assays. Here, flowers demonstrated the strongest metal-binding capacity (2.44 mg/mL), while stems, leaves, and bracts clustered between 3.91–4.88 mg/mL. Roots showed minimal chelating efficiency with an IC_50_ of 11.96 mg/mL, markedly weaker than EDTA (0.020 mg/mL). Unlike the electron-transfer and radical-scavenging assays, ferrous ion chelation reflects the ability of compounds to bind transition metals rather than to directly neutralize reactive species; therefore, it represents a related but distinct aspect of antioxidant behavior.

**Figure 2:**
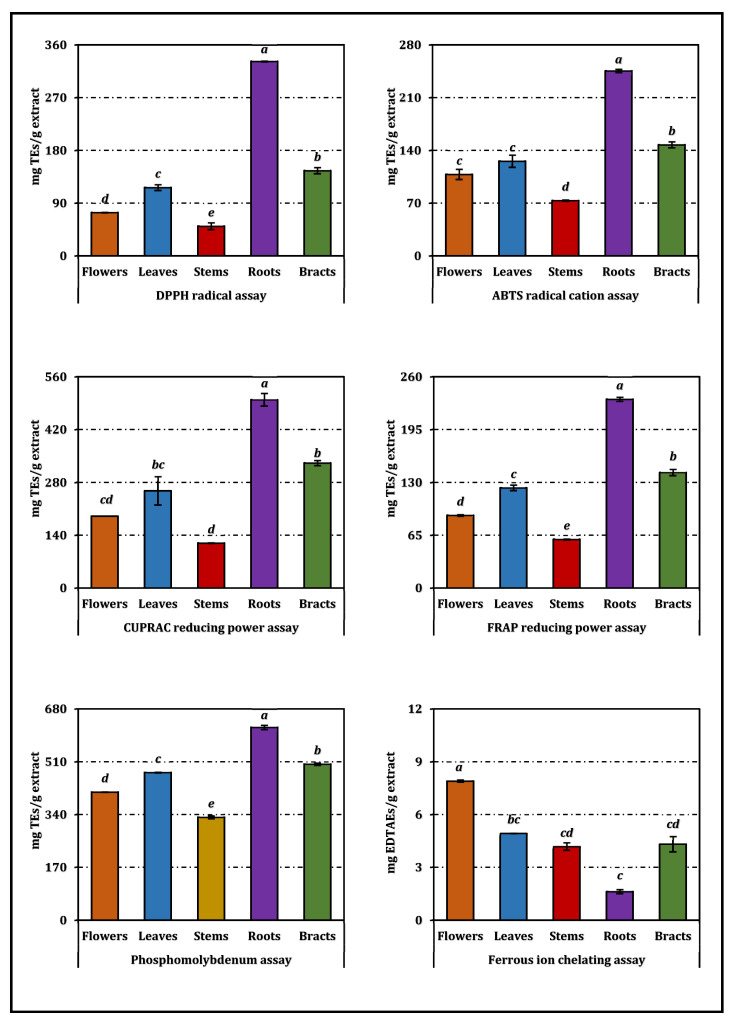
Comparative visualization of the antioxidant-related assays for *A. dioscoridis* var. *perringii* extracts, including electron-transfer/radical-scavenging assays and ferrous ion chelating activity. TEs and EDTAEs, Trolox, and ethylenediaminetetraacetic acid (disodium salt) equivalents, respectively. Values indicated by the same superscripts (a–e) on the bar chart were not significantly different according to Tukey’s HSD test at the 5% significance level.

**Figure 3:**
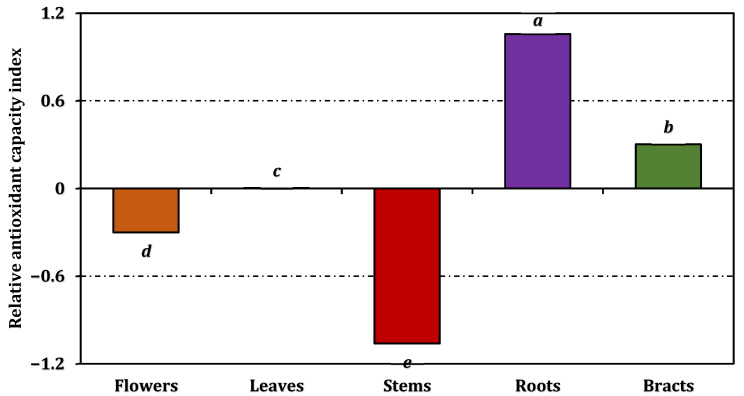
Relative antioxidant capacity index of *A. dioscoridis* var. *perringii* extracts. Values indicated by the same superscripts (a–e) on the bar chart were not significantly different according to Tukey’s HSD test at the 5% significance level.

**Figure 4:**
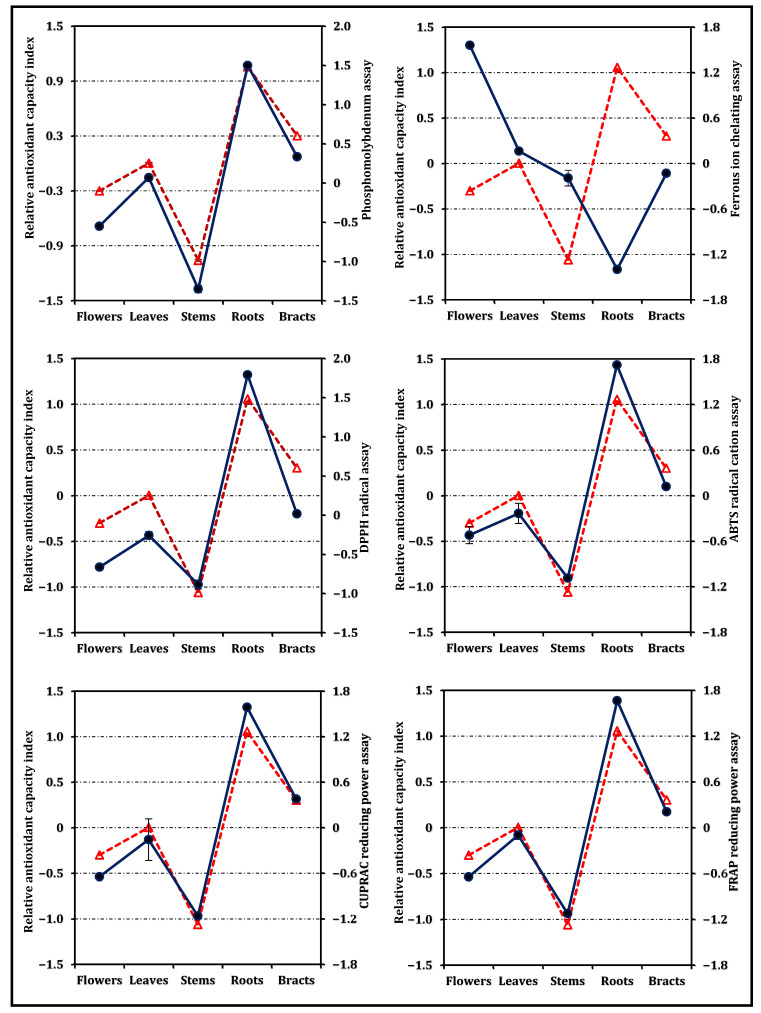
Graphical comparison between the Relative Antioxidant Capacity Index (RACI) and the trends of the antioxidant activity indices obtained from different assays. The dashed red line (triangle markers) represents the RACI values, while the solid blue line (circle markers) represents the normalized trends of the antioxidant activity indices derived from individual assays.

Results from the antioxidant-related assays—encompassing both electron-transfer tests and the metal-chelating assay—are presented in Figure 2. The RACI results (Figure 3) confirmed the overall antioxidant ranking of the organs, with roots showing the strongest integrated antioxidant performance, followed by bracts and leaves. Building upon these findings, a graphical comparison of RACI values with antioxidant activity trends (Figure 4) demonstrated consistent patterns between RACI and electron-transfer-based assays (phosphomolybdenum, CUPRAC, FRAP, DPPH, and ABTS). In contrast, ferrous ion chelating activity exhibited inverse trends relative to RACI for flowers, stems, and roots, reflecting the opposite response direction of this assay compared to electron-transfer-based methods. However, the trends shown in Figure 4 and the associated comparative evaluation were based on normalized antioxidant activity values derived from the experimental dataset, rather than directly on the IC_50_/EC_50_ values.

It should be noted that the IC_50_ and EC_50_ values reported in this study were generated from concentration–response assays prepared directly from the crude extracts; therefore, they describe the activity of the obtained extracts rather than the activity recalculated per unit dry plant material. All bioactivity assays were performed based on extract concentration (mg/mL), and therefore the results reflect extract-based activity and were not normalized to the dry weight of the plant material. Because extraction yields differed among organs (Section 3.3), a lower IC_50_ may reflect not only stronger extract activity but also differences in extract recovery from the original tissue.

In principle, the results could be further converted to a dry-weight basis to estimate activity relative to the starting plant material. However, this was not the primary analytical framework of the present study, which was designed to compare organ extracts as experimentally obtained and tested under the same assay conditions. For this reason, the IC_50_/EC_50_ data are presented on an extract basis, while extraction yield is provided separately to support a more cautious interpretation of organ-to-organ differences.

The present study provides the first organ-specific assessment of antioxidant properties in *A. dioscoridis* var. *perringii*, thus extending the phytochemical and bioactivity knowledge of the genus *Acanthus*. The consistently comparatively stronger performance of the root extract in all electron-transfer-based assays suggests a distinctive antioxidant profile within this variety, setting it apart from the limited but informative data available for *A. dioscoridis* var. *dioscoridis*. In the latter taxon, methanolic extracts also exhibited pronounced radical-scavenging and reducing capacities, markedly outperforming non-polar fractions [7]. This parallel suggests that polar phenolic constituents play a central role in shaping antioxidant responses across both varieties, even though the magnitude and organ distribution observed in the present study are unique to var. *perringii*.

Comparisons with other *Acanthus* taxa further contextualize these findings. Several species, including *A. ilicifolius*, *A. ebracteatus*, and *A. mollis*, have been reported to exhibit strong antioxidant activity, particularly in polar extracts enriched with phenolics [19,20,21,22]. For instance, *A. ebracteatus* leaves display exceptionally high reducing and radical-scavenging capacities, accompanied by substantial phenolic and flavonoid levels [21]. Similarly, methanol and ethanol extracts of *A. ilicifolius* consistently demonstrate potent radical-scavenging activity, with methanolic flower extracts notably achieving the highest antiradical efficiency in their respective studies [23]. This recurrent pattern across the genus aligns with the strong electron-transfer activity detected in *A. dioscoridis* var. *perringii* roots, suggesting that these organs may contain relatively higher levels of phenolic or other redox-active constituents, although direct causal relationships cannot be confirmed from the present data.

The striking divergence observed in metal chelation, where flowers—rather than roots—showed the strongest activity, mirrors reports from var. *dioscoridis*, in which non-polar fractions such as hexane perform best in chelating assays [7]. This supports the notion that metal-binding capacity in the genus is not strictly phenolic-driven but may depend on distinct ligand groups with an affinity for transition metals. The inverse correlation between the chelating data and electron-transfer assays in the present study reinforces this mechanistic distinction and has also been observed in other *Acanthus* species where ET-based antioxidant measures align strongly with phenolic levels, unlike chelation [22].

The positive correlations between RACI and all electron-transfer assays are consistent with the broader *Acanthus* literature, where ET-based responses tend to co-vary due to shared dependency on redox-active constituents [8,22]. The fact that the roots of var. *perringii* achieved the highest composite score suggests a functional clustering of ET-based mechanisms in this organ. The organ-specific nature of this profile contrasts with species such as *A. mollis*, where antioxidant activity does not vary significantly among organs [22], and underscores the biological distinctiveness of *A. dioscoridis* var. *perringii*.

Overall, the antioxidant data show a clear organ-dependent pattern in *A. dioscoridis* var. *perringii*, with roots standing out as the most active organ in most electron-transfer and radical-scavenging assays.

### 2.3. Enzyme Inhibitory Activity

The extracts of *A. dioscoridis* var. *perringii* exhibited organ-dependent inhibitory effects across all tested enzymes (Table 3). In the cholinesterase assays, AChE inhibition showed a relatively narrow IC_50_ range (1.00–1.05 mg/mL), indicating only minor variation among plant organs, although the stem extract displayed the lowest IC_50_ value. A similar pattern was observed for BChE, although inhibition varied more distinctly among organs; the stems again demonstrated the lowest IC_50_ value (1.06 mg/mL), while the roots and bracts were less potent. All extracts, as expected, remained markedly weaker than the reference inhibitor galanthamine.

Tyrosinase inhibition was modest and displayed limited variation among flower, leaf, root, and bract extracts (IC_50_ ≈ 1.15–1.16 mg/mL), suggesting that organ-specific differences are minimal for this enzyme. The stems, however, showed slightly reduced efficacy (1.18 mg/mL). None of the plant-derived samples approached the activity of kojic acid.

The carbohydrate-hydrolyzing enzymes revealed greater separation among organs. For α-amylase, the leaves produced the lowest IC_50_ value (3.46 mg/mL), followed by the flowers, bracts, and roots, whereas stems exhibited the weakest inhibition (5.67 mg/mL). In contrast, α-glucosidase inhibition clustered more tightly, with bracts and roots yielding the most potent activity (0.97–0.99 mg/mL), comparable to the stem extract (1.00 mg/mL). All extracts were generally less active than acarbose.

**Figure 5:**
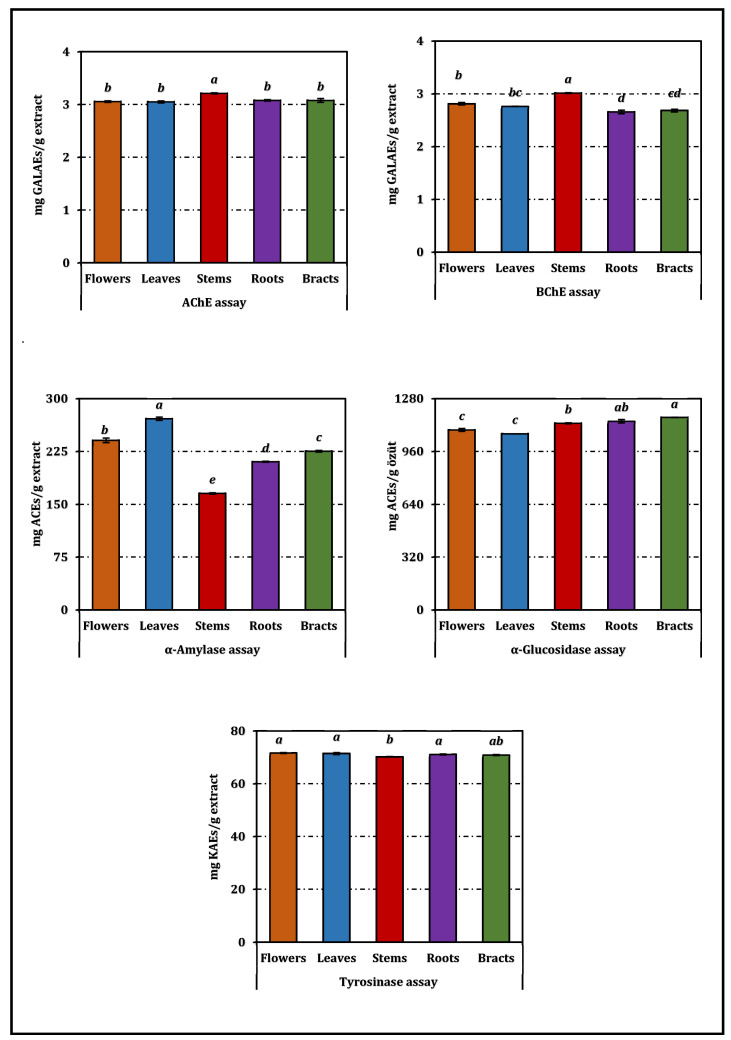
Enzyme inhibition activity of *A. dioscoridis* var. *perringii* extracts. ACEs, GALAEs and KAEs mean acarbose, galanthamine and kojic acid equivalents, respectively. Values indicated by the same superscripts (a–e) on the bar chart are not significantly different according to Tukey’s HSD test at the 5% significance level.

The enzyme inhibition results are presented primarily in Table 3, which provides the exact IC_50_ values used for interpretation. For completeness, the corresponding positive-control-equivalent visualization is presented in Figure 5.

Overall, the enzyme inhibition results indicate moderate but organ-dependent differences, with Table 3 providing the principal basis for comparison.

The literature data on *Acanthus* species, particularly the reported enzyme-modulatory relevance of verbascoside and related metabolites [24,25,26], support the interpretation of the observed organ-level differences in the present study.

In the context of diabetes-related enzyme inhibition, the α-amylase and α-glucosidase inhibitory activity patterns observed in *A. dioscoridis* var. *perringii* also reflect established trends within the genus. *A. montanus* leaf extracts have been reported to inhibit both enzymes, with methanol extracts displaying superior activity and reported inhibition modes of non-competitive behavior for α-amylase and competitive behavior for α-glucosidase [27]. Similarly, extracts of *A. ilicifolius* have shown a notable inhibition of both enzymes, reinforcing the view that *Acanthus* species may serve as natural modulators of postprandial glucose metabolism [28]. Although enzyme inhibition in *A. dioscoridis* var. *perringii* was comparatively weaker than in these species, the organ-dependent variation—particularly the relatively stronger α-amylase and α-glucosidase inhibition in the leaf, bract, and root extracts—provides useful information on the biochemical distribution of bioactivity across plant organs. Given that the IC_50_ values were in the milligram-per-milliliter range, these activities should be regarded as moderate to weak when compared with standard inhibitors, and therefore mainly informative for comparative phytochemical and biochemical characterization rather than for pharmacological development.

The broader pattern across *Acanthus* species indicates that enzyme inhibitory potential is often associated with phenylethanoid glycosides, benzoxazinoids, and other specialized metabolites. This is exemplified by the BChE, α-amylase, and α-glucosidase inhibitory effects reported for *A. spinosus*, whose EtOAc extract exhibited selective potency reflective of its enriched phenolic profile [29]. Thus, while the overall inhibitory strength of *A. dioscoridis* var. *perringii* remains modest, its phytochemical context and organ-specific trends contribute to the comparative biochemical characterization of Acanthus species and help map the distribution of phenolic-related bioactivities within the genus.

In summary, the enzyme inhibition findings primarily contribute to the biochemical profiling of *A. dioscoridis* var. *perringii* and highlight organ-dependent variation rather than strong pharmacological differentiation.

### 2.4. Correlations Among Phenolic Compounds and Assays

The correlation results were interpreted as statistical associations between compound abundance and bioactivity parameters.

Correlations among phenolic compounds and assays are summarized in Table 4. Because RACI represents a standardized composite index derived from several antioxidant assays, its relationships with individual tests were examined to determine whether the integrated index reflects the same antioxidant trends observed in the electron-transfer assays. Total antioxidant capacity (TAP) showed very strong positive associations with all electron-transfer-based assays and the composite RACI (*r* = 0.944–0.996), indicating that extracts performing well in one antioxidant test generally performed well across the entire panel. In line with this, the total phenolic content was almost perfectly correlated with FRAP (*r* = 0.997), ABTS (*r* = 0.993), CUPRAC (*r* = 0.992), and TAP (*r* = 0.990), suggesting that phenolic compounds may contribute substantially to the global antioxidant profile. In contrast, ferrous ion chelating activity (FICA) was moderately to strongly and inversely related to these parameters (*r* = −0.618 to −0.740 for TAP, DPPH, ABTS, CUPRAC, and FRAP), suggesting a partly distinct chelation-driven mechanism compared with the predominantly electron-transfer-based assays.

Cholinesterase inhibition showed a close association with the antioxidant matrix, whereas tyrosinase and α-amylase inhibition were more closely related to selected phenolic constituents (Table 4).

Overall, the correlation analysis supports a relationship between phenolic richness and antioxidant/cholinesterase responses while suggesting a more selective contribution of certain phenolic acids and flavonoid derivatives to metal chelation and carbohydrase-related inhibition.

## 3. Materials and Methods

### 3.1. Chemicals

Gallic acid, (+)-catechin, pyrocatechol, chlorogenic acid, 2,5-dihydroxybenzoic acid, 4-hydroxybenzoic acid, (−)-epicatechin, caffeic acid, syringic acid, vanillin, taxifolin, sinapic acid, *p*-coumaric acid, ferulic acid, rosmarinic acid, 2-hydroxycinnamic acid, pinoresinol, quercetin, luteolin, and apigenin were purchased from Sigma-Aldrich (St. Louis, MO, USA). Vanillic acid, 3-hydroxybenzoic acid, 3,4-dihydroxyphenylacetic acid, apigenin 7-glucoside, luteolin 7-glucoside, hesperidin, eriodictyol, and kaempferol were obtained from Fluka (St. Louis, MO, USA). Finally, verbascoside, protocatechuic acid, and hyperoside were purchased from HWI Analytik (Rülzheim, Germany).

Folin–Ciocalteu reagent, aluminum trichloride (AlCl_3_), 1,1-diphenyl-2-picrylhydrazyl (DPPH), ABTS (2,2′-azinobis(3-ethylbenzothiazoline-6-sulfonic acid)), potassium persulfate, ferrozine, neocuproine, 2,4,6-tris(2-pyridyl)-s-triazine (TPTZ), α-amylase (from porcine pancreas), α-glucosidase (from Saccharomyces cerevisiae), tyrosinase (from mushroom), acetylcholinesterase (from *Electrophorus electricus*), and butyrylcholinesterase (from equine serum), p-nitrophenyl-α-D-glucopyranoside (PNPG), L-DOPA, 5,5′-dithiobis-(2-nitrobenzoic acid) (DTNB), acetylthiocholine iodide (ATCI), butyrylthiocholine chloride (BTCl), Trolox, galanthamine, kojic acid, and acarbose were obtained from Sigma-Aldrich (St. Louis, MO, USA).

Sodium carbonate (Na_2_CO_3_), sulfuric acid, sodium phosphate, ammonium molybdate, ferrous chloride (FeCl_2_), cupric chloride (CuCl_2_), ammonium acetate, ferric chloride (FeCl_3_), hydrochloric acid, starch, glutathione, and ethylenediaminetetraacetic acid disodium salt (EDTA) were purchased from Merck (Darmstadt, Germany).

Methanol and formic acid of HPLC grade were purchased from Sigma-Aldrich (St. Louis, MO, USA) and Merck (Darmstadt, Germany), respectively. Ultra-pure water (18 MΩ) was obtained using a Millipore Milli-Q Plus water purification system (Millipore Bedford Corp., Bedford, MA, USA).

### 3.2. Plant Material

Specimens of *A. dioscoridis* var. *perringii* were collected at full flowering on 1 August 2025 from gypsum-rich calcareous soils located in Kümbet Village, Zara District, Sivas, Turkey (39°48′20″ N, 37°49′02″ E; 1895 m altitude). The taxonomic identification was performed by Dr. Bedrettin Selvi, and a voucher specimen (GOPU 9618) was deposited in the Herbarium of the Faculty of Arts and Sciences, Tokat Gaziosmanpaşa University. Five plant organs—leaves, stems, flowers, roots, and bracts—were individually sampled and processed. Following collection, each organ was separated, air-dried under shaded and well-ventilated conditions for several weeks until a constant weight was reached, and subsequently ground into a fine powder using a laboratory mill prior to analysis. Accordingly, the dry weight of the starting material was defined as the constant mass of each organ recorded after the air-drying step and immediately before extraction. Plant material from several individuals collected at the same site and sampling date was pooled prior to processing in order to obtain a representative organ-level sample. The extraction procedure was performed once for each pooled organ sample. Accordingly, the study did not include independent biological replicates at the organ level; instead, each organ extract represented a pooled sample, and subsequent assay repetitions corresponded to technical replicates of that pooled extract.

### 3.3. Ethanol Extraction

Powdered plant materials of each organ (flowers, leaves, stems, roots, and bracts), prepared as described in Section 3.1, were used for ethanol extraction. Ethanol extraction was performed for one hour using an ultrasound-assisted extraction (UAE) approach in a sonication bath with a constant sample-to-solvent ratio of 1:20 [30,31]. For each organ, 5 g of the previously dried plant material (expressed on a dry-weight basis) was extracted with 100 mL of absolute ethanol in an ultrasonic bath operated at 40 kHz and 260 W at approximately 30 °C for 60 min. After extraction, the mixtures were filtered through Whatman No. 1 filter paper, and the solvent was removed under reduced pressure using a rotary evaporator. After evaporation, each extract was dried further until a constant weight was obtained in order to eliminate residual solvent. The dry weight of the extract was then defined as the constant mass of the solvent-free residue measured gravimetrically. Extraction yield was calculated by dividing this dry extract mass by the dry weight of the starting plant material taken for extraction and expressing the result as percentage (*w*/*w*). The dried extracts were then stored at 4 °C until further experiments.

Extraction yields for the flowers, leaves, stems, roots, and bracts of *A. dioscoridis* var. *perringii* were 4.92%, 3.78%, 5.60%, 6.12%, and 5.30%, respectively, and were calculated as follows:

Extract yield (%) = (constant dry mass of the extract obtained after evaporation and final solvent removal/constant dry mass of the starting plant material used for extraction) × 100.

These values are expressed as % extract yield relative to the dry weight of plant material (*w*/*w*). Thus, both the starting material and the recovered extracts were handled on a dry-weight basis for yield calculation, whereas the subsequent antioxidant and enzyme inhibition assays were performed using the dried crude extracts re-dissolved at the required test concentrations. Accordingly, the resulting IC_50_/EC_50_ values represent extract-based activity, whereas yield information indicates how much extract was recovered from a given dry mass of plant material.

### 3.4. Determination of the Chemical Compositions of the Extracts

Total phenolic content was determined using the Folin–Ciocalteu reagent and expressed as gallic acid equivalents, while the total flavonoid content was quantified using the aluminum chloride colorimetric method and expressed as rutin equivalents [32,33]. The phytochemical profiles of the extracts were determined using a previously validated LC-ESI-MS/MS method for the simultaneous quantification of phenolic compounds [34]. Quantitative analyses were performed using an Agilent Technologies 1260 Infinity liquid chromatography system (Agilent Technologies, Santa Clara, CA, USA) coupled to a 6420 Triple Quad mass spectrometer (Agilent Technologies, Santa Clara, CA, USA). Chromatographic separation was achieved on a Poroshell 120 EC-C18 column (100 mm × 4.6 mm I.D., 2.7 μm) using a mobile phase consisting of solvent A (0.1% formic acid in water) and solvent B (methanol) under a gradient elution program. The column temperature was maintained at 25 °C, the flow rate was set to 0.4 mL min^−1^, and the injection volume was 2 μL. Detection was carried out using an electrospray ionization (ESI) source operating in multiple reaction monitoring (MRM) mode in both negative and positive ionization modes. Phenolic compounds were identified and quantified by comparing their retention times and characteristic ion transitions with those of authentic standards. The analytical performance characteristics of this method are provided in Tables S1 and S2 of the Supplementary Materials.

### 3.5. Biological Activity

Antioxidant and enzyme inhibition activities of the extracts were evaluated using a panel of widely applied spectrophotometric assays. Total antioxidant capacity was additionally evaluated using the phosphomolybdenum method. Radical scavenging capacity was assessed using the DPPH and ABTS assays, whereas the reducing power was measured using CUPRAC and ferric reducing antioxidant power (FRAP) assays. The metal chelating capacity of the extracts was determined using the ferrozine–Fe^2+^ complex formation method.

Enzyme inhibitory activities were evaluated against several metabolically relevant enzymes. The inhibitory effects on α-amylase and α-glucosidase were determined using starch–iodine and PNPG-based colorimetric assays, respectively. Tyrosinase inhibition was assessed using a modified dopachrome method with L-DOPA as the substrate, whereas cholinesterase inhibitory activity against acetylcholinesterase (AChE) and butyrylcholinesterase (BuChE) was determined according to Ellman’s method. For enzyme inhibition, radical scavenging, and the metal chelation assays, IC_50_ values were calculated as the extract concentration required to inhibit the initial activity by 50%, while EC_50_ values were defined as the extract concentration providing an absorbance of 0.500 for reducing power and phosphomolybdenum assays.

The assays were carried out using previously reported protocols [31,32,35,36,37,38,39], and detailed experimental conditions are provided in the Supplementary Materials.

IC_50_ (or EC_50_, where applicable) values were obtained from concentration–response data by plotting the extract concentration against percentage inhibition/activity. For antioxidant activity assays, IC_50_ (or EC_50_, where applicable) values were calculated using linear regression analysis, whereas for enzyme inhibition assays, IC_50_ values were determined using nonlinear curve fitting (four-parameter logistic regression model) to estimate the concentration required to produce 50% effect.

### 3.6. Statistical Analysis

All data were expressed as mean ± standard deviation (SD). Each assay was performed in triplicate (*n* = 3) for each organ extract, representing technical replicates of extract-based measurements derived from the same plant material. No independent biological replicate extraction series were performed, because each organ type was represented by a single pooled sample prepared from multiple individuals collected at one site and one sampling time. Statistical comparisons were performed using one-way ANOVA followed by Tukey’s post hoc test, with *p* < 0.05 considered statistically significant. Analyses were conducted using SPSS version 26.0.

Pearson’s correlation analysis was applied to evaluate the relationships among variables, and the statistical significance of the correlation coefficients was assessed using corresponding *p*-values. Because the number of extract samples used in the correlation analysis was limited (*n* = 5), Spearman rank correlation coefficients were additionally calculated as a non-parametric robustness check, and the results are presented in the Supplementary Materials (Supplementary Table S3).

Due to differences in the underlying mechanisms of the antioxidant assays, a direct comparison of the raw outcomes was not appropriate. Therefore, the Relative Antioxidant Capacity Index (RACI) was calculated to standardize the results across assays. RACI values were obtained by subtracting the assay-specific mean from the raw values and dividing by the corresponding standard deviation [1], according to the equation:

RACI = (x − μ)/σ,

where *x* is the raw assay value, μ is the mean value of the assay, and σ is the standard deviation. This z-score transformation allows antioxidant assays with different measurement scales to be integrated into a single comparative metric without bias toward assays producing numerically larger values. Because RACI is based on z-score standardization, negative values do not imply the absence or reversal of antioxidant activity. Rather, they indicate that the corresponding sample exhibits a lower antioxidant performance relative to the mean of the dataset for that assay, while positive values indicate above-average performance. Accordingly, RACI values should be interpreted as relative, dimensionless indices used for comparative ranking across different assays.

For the calculation of RACI values, the raw data (x) corresponded to the normalized antioxidant activity values used for graphical comparison in Figure 4, rather than directly to the IC_50_ or EC_50_ values presented in Table 2. These normalized values were derived from the experimental antioxidant assay results by transforming the data into a common scale to enable comparability across methods with different units and response directions.

For each assay, the mean value (μ) and standard deviation (σ) were calculated using the dataset comprising all five organ extracts (flowers, leaves, stems, roots, and bracts), and these parameters were then used to standardize each measurement according to the RACI equation. Thus, the RACI values represent standardized scores derived exclusively from the experimental antioxidant activity data generated in this study, ensuring consistency between the graphical representation and the comparative evaluation.

In addition, the agreement between the RACI trends and individual antioxidant assays was evaluated to examine the consistency of the composite index with the underlying antioxidant responses.

### 3.7. Use of Artificial Intelligence

Artificial intelligence (AI) tools were utilized exclusively to support language refinement, conceptual clarity, and overall presentation quality of the manuscript. All experimental procedures, laboratory analyses, and data generation were conducted entirely by the authors without AI involvement. The contribution of AI was limited to improving textual coherence, readability, and technical precision. All AI-assisted components were thoroughly reviewed and approved by the authors to ensure full compliance with scientific rigor, transparency, and research integrity.

## 4. Conclusions

This study shows a clear organ-dependent distribution of phenolic compounds and related bioactivities in *A. dioscoridis* var. *perringii*. Roots were the richest phenolic organ and showed the strongest overall antioxidant performance in most assays, whereas bracts had the highest flavonoid content, flowers showed the strongest metal-chelating capacity, stems exhibited the strongest cholinesterase inhibition, and leaves and bracts were relatively more active against α-amylase and α-glucosidase, respectively.

These findings provide the first organ-level phytochemical and bioactivity profile of this taxon and clarify how major phenolic traits vary among plant organs. In applied terms, they help identify the most relevant organs for future targeted phytochemical and bioactivity-oriented studies. Because the work is based on crude extracts from a single population and in vitro assays, the results should be regarded as a foundation for future compound isolation, targeted validation studies, and broader comparative analyses.

## Figures and Tables

**Table 1:** Concentration (µg/g extract) of selected phenolic compounds in the *A. dioscoridis* var. *perringii* extracts.

Compounds	Flowers	Leaves	Stems	Roots	Bracts
Verbascoside	12,382 ± 134 *^c^*	16,641 ± 144 *^a^*	11,655 ± 129 *^d^*	16,390 ± 171 *^a^*	15,551 ± 4 *^b^*
Protocatechuic acid	86.4 ± 0.1 *^b^*	225 ± 5 *^a^*	24.5 ± 0.1 *^c^*	18.6 ± 0.1 *^c^*	87.1 ± 0.2 *^b^*
Hesperidin	158 ± 3 *^c^*	210 ± 1 *^b^*	31.5 ± 0.1 *^d^*	9.27 ± 0.15 *^e^*	264 ± 3 *^a^*
3-Hydroxybenzoic acid	327 ± 2 *^a^*	209 ± 1 *^b^*	23.6 ± 0.2 *^d^*	8.77 ± 0.13 *^e^*	156 ± 2 *^c^*
4-Hydroxybenzoic acid	325 ± 5 *^a^*	208 ± 4 *^b^*	21.8 ± 1.1	8.19 ± 0.14	152 ± 1 *^c^*
Luteolin 7-glucoside	104 ± 2 *^c^*	183 ± 2 *^a^*	40.6 ± 1.2 *^d^*	2.25 ± 0.13 *^e^*	169 ± 1 *^b^*
Syringic acid	74.9 ± 0.8 *^c^*	94.4 ± 0.7 *^a^*	84.4 ± 0.2 *^b^*	55.9 ± 0.4 *^d^*	26.7 ± 0.7 *^e^*
Hyperoside	74.5 ± 2.2 *^a^*	53.8 ± 0.7 *^b^*	10.7 ± 0.1 *^c^*	9.12 ± 0.09 *^c^*	54.8 ± 1.1 *^b^*
Luteolin	222 ± 5 *^a^*	44.9 ± 0.2 *^c^*	11.7 ± 0.2 *^d^*	1.01 ± 0.06 *^e^*	169 ± 1 *^b^*
p-Coumaric acid	56.3 ± 0.8 *^a^*	35.9 ± 0.2 *^b^*	22.8 ± 0.5 *^d^*	19.7 ± 0.5 *^e^*	29.0 ± 0.5 *^c^*
Ferulic acid	37.8 ± 0.7 *^a^*	19.6 ± 0.6 *^b^*	16.9 ± 0.2 *^c^*	9.01 ± 0.03 *^e^*	14.9 ± 0.1 *^d^*
Apigenin 7-glucoside	42.7 ± 1.4 *^b^*	18.8 ± 0.2 *^c^*	7.75 ± 0.27 *^d^*	2.53 ± 0.03 *^e^*	72.3 ± 0.8 *^a^*
Chlorogenic acid	53.2 ± 0.4 *^a^*	17.0 ± 0.1 *^b^*	13.3 ± 0.1 *^d^*	16.0 ± 0.3 *^c^*	14.2 ± 0.1 *^d^*
Caffeic acid	33.1 ± 0.5 *^a^*	12.0 ± 0.3 *^d^*	13.7 ± 0.3 *^c^*	15.7 ± 0.3 *^b^*	16.7 ± 0.1 *^b^*
Apigenin	97.2 ± 1.7 *^a^*	12.0 ± 0.1 *^c^*	7.06 ± 0.01 *^d^*	nd	63.1 ± 0.8 *^b^*
Vanillin	44.8 ± 0.5 *^a^*	11.2 ± 0.1 *^c^*	7.96 ± 0.08 *^d^*	11.2 ± 0.1 *^c^*	22.9 ± 0.2 *^b^*
Rosmarinic acid	8.00 ± 0.45 *^b^*	9.02 ± 0.12 *^a^*	5.82 ± 0.02 *^c^*	5.03 ± 0.13 *^c^*	5.32 ± 0.04 *^c^*
Sinapic acid	18.7 ± 0.1 *^a^*	7.75 ± 0.35 *^b^*	2.92 ± 0.10 *^d^*	1.60 ± 0.06 *^e^*	6.21 ± 0.01 *^c^*
Gallic acid	5.74 ± 0.05 *^a^*	5.10 ± 0.16 *^b^*	4.36 ± 0.09 *^c^*	5.91 ± 0.02 *^a^*	5.34 ± 0.05 *^b^*
Quercetin	10.5 ± 0.2 *^a^*	4.41 ± 0.14 *^c^*	3.97 ± 0.04 *^c^*	2.78 ± 0.14 *^d^*	5.99 ± 0.15 *^b^*
(−)-Epicatechin	nd	nd	nd	nd	nd
(+)-Catechin	nd	nd	nd	nd	nd
2-Hydroxycinnamic acid	nd	nd	nd	nd	nd
3,4-Dihydroxyphenylacetic acid	nd	nd	nd	nd	nd
Eriodictyol	nd	nd	nd	nd	nd
Kaempferol	nd	nd	nd	nd	nd
Pinoresinol	nd	nd	nd	nd	nd
Taxifolin	nd	nd	nd	nd	nd

Values indicated by the same superscripts (a–e) within the same row were not significantly different according to Tukey’s HSD test at the 5% significance level. nd: Not detected.

**Table 2:** Antioxidant and metal-chelating activities of *A. dioscoridis* var. *perringii* extracts.

Assays	Flowers	Leaves	Stems	Roots	Bracts	Trolox	EDTA
Phosphomolybdenum (EC_50_: mg/mL)	1.08 ± 0.002 *^d^*	0.94 ± 0.002 *^c^*	1.34 ± 0.02 *^e^*	0.72 ± 0.01 *^b^*	0.89 ± 0.01 *^c^*	0.45 ± 0.03 *^a^*	
CUPRAC reducing power (EC_50_: mg/mL)	0.91 ± 0.001 *^d^*	0.68 ± 0.10 *^c^*	1.45 ± 0.01 *^e^*	0.35 ± 0.01 *^b^*	0.52 ± 0.01 *^c^*	0.17 ± 0.01 *^a^*	
FRAP reducing power (EC_50_: mg/mL)	0.55 ± 0.01 *^e^*	0.40 ± 0.01 *^d^*	0.82 ± 0.01 *^e^*	0.21 ± 0.01 *^b^*	0.35 ± 0.01 *^c^*	0.049 ± 0.003 *^a^*	
DPPH radical (IC_50_: mg/mL)	3.43 ± 0.02 *^d^*	2.18 ± 0.09 *^c^*	5.05 ± 0.60 *^e^*	0.77 ± 0.002 *^ab^*	1.75 ± 0.06 *^bc^*	0.24 ± 0.02 *^a^*	
ABTS radical cation (IC_50_: mg/mL)	1.62 ± 0.10 *^d^*	1.39 ± 0.09 *^cd^*	2.38 ± 0.03 *^e^*	0.71 ± 0.01 *^b^*	1.19 ± 0.03 *^c^*	0.16 ± 0.03 *^a^*	
Ferrous ion chelating (IC_50_: mg/mL)	2.44 ± 0.02 *^ab^*	3.91 ± 0.01 *^b^*	4.63 ± 0.24 *^b^*	11.96 ± 0.85 *^c^*	4.88 ± 0.98 *^b^*		0.020 ± 0.001 *^a^*

EDTAE mean ethylenediaminetetraacetic acid (disodium salt). Values indicated by the same superscripts (a–e) within the same row were not significantly different according to Tukey’s HSD test at the 5% significance level.

**Table 3:** Enzyme inhibition activity of *A. dioscoridis* var. *perringii* extracts.

Samples	AChE Inhibition (IC_50_: mg/mL)	BChE Inhibition (IC_50_: mg/mL)	Tyrosinase Inhibition (IC_50_: mg/mL)	α-Amylase Inhibition (IC_50_: mg/mL)	α-Glucosidase Inhibition (IC_50_: mg/mL)
Flowers	1.05 ± 0.01 *^c^*	1.14 ± 0.01 *^c^*	1.15 ± 0.003 *^b^*	3.90 ± 0.05 *^c^*	1.04 ± 0.01 *^bc^*
Leaves	1.05 ± 0.01 *^c^*	1.16 ± 0.001 *^c^*	1.15 ± 0.01 *^b^*	3.46 ± 0.03 *^b^*	1.06 ± 0.001 *^c^*
Stems	1.00 ± 0.003 *^b^*	1.06 ± 0.002 *^b^*	1.18 ± 0.001 *^c^*	5.67 ± 0.03 *^f^*	1.00 ± 0.002 *^ab^*
Roots	1.04 ± 0.005 *^c^*	1.20 ± 0.02 *^d^*	1.16 ± 0.003 *^b^*	4.46 ± 0.01 *^e^*	0.99 ± 0.01 *^ab^*
Bracts	1.04 ± 0.01 *^c^*	1.19 ± 0.01 *^d^*	1.16 ± 0.003 *^b^*	4.17 ± 0.02 *^d^*	0.97 ± 0.001 *^a^*
Galanthamine	0.0031 ± 0.0003 *^a^*	0.0031 ± 0.0002 *^a^*			
Kojic acid			0.082 ± 0.002 *^a^*		
Acarbose				0.93 ± 0.04 *^a^*	1.12 ± 0.03 *^d^*

Values indicated by the same superscripts (a–f) within the same column were not significantly different according to Tukey’s HSD test at the 5% significance level.

**Table 4:** Correlations among phenolic compounds and assays.

	TAP	DPPH	ABTS	CUPRAC	FRAP	FICA	AChEIA	BChEIA	TIA	AAIA	AGIA
DPPH radical	0.944										
ABTS radical cation	0.970	0.990									
CUPRAC reducing power	0.984	0.975	0.981								
FRAP reducing power	0.984	0.986	0.994	0.989							
Ferrous ion chelating	−0.618	−0.740	−0.671	−0.695	−0.697						
RACI	0.996	0.933	0.966	0.975	0.975						
AChE inhibition	−0.594	−0.326	−0.431	−0.481	−0.456	−0.177					
BChE inhibition	−0.923	−0.765	−0.834	−0.861	−0.853	0.340	0.841				
Tyrosinase inhibition	0.413	0.176	0.275	0.313	0.278	0.415	−0.921	−0.655			
α-Amylase inhibition	0.321	0.027	0.122	0.174	0.168	0.373	−0.876	−0.576	0.864		
α-Glucosidase inhibition	0.265	0.419	0.369	0.387	0.349	−0.677	0.349	−0.069	−0.518	−0.690	
Total flavonoid	0.606	0.326	0.406	0.502	0.469	−0.168	−0.822	−0.787	0.595	0.746	−0.085
Total phenolic	0.990	0.979	0.993	0.992	0.997	−0.647	−0.506	−0.877	0.344	0.211	0.322
Verbascoside	0.839	0.689	0.715	0.771	0.777	−0.543	−0.630	−0.825	0.409	0.571	−0.047
Protocatechuic acid	−0.022	−0.289	−0.233	−0.161	−0.161	0.358	−0.540	−0.193	0.530	0.855	−0.780
Hesperidin	−0.085	−0.402	−0.308	−0.209	−0.254	0.453	−0.628	−0.245	0.548	0.745	−0.423
3-Hydroxybenzoic acid	−0.239	−0.486	−0.377	−0.360	−0.379	0.873	−0.612	−0.113	0.740	0.722	−0.670
4-Hydroxybenzoic acid	−0.238	−0.483	−0.374	−0.358	−0.377	0.875	−0.613	−0.113	0.746	0.724	−0.679
Luteolin 7-glucoside	−0.171	−0.473	−0.399	−0.301	−0.331	0.452	−0.526	−0.124	0.472	0.761	−0.577
Syringic acid	−0.529	−0.505	−0.532	−0.577	−0.521	0.464	0.262	0.534	−0.040	0.171	−0.796
Hyperoside	−0.182	−0.459	−0.346	−0.307	−0.336	0.795	−0.661	−0.183	0.730	0.746	−0.580
Luteolin	−0.227	−0.414	−0.308	−0.293	−0.337	0.748	−0.486	−0.103	0.565	0.389	−0.209

Data show the Pearson Correlation Coefficients between the parameters. TAP: total antioxidant activity by phosphomolybdenum method. AAIA, AGIA, AChEIA, BChEIA and TIA: α-amylase, α-glucosidase, acetylcholinesterase, butyrylcholinesterase and tyrosinase inhibition activities, respectively. ABTS and DPPH: ABTS and DPPH radical scavenging activities, respectively. CUPRAC and FRAP: cupric ion reducing antioxidant capacity and ferric reducing antioxidant power; respectively. FICA: Ferrous ion chelating activity. RACI: Relative antioxidant capacity index.

## Data Availability

The original contributions presented in this study are included in the article/Supplementary Materials. Further inquiries can be directed to the corresponding author.

## Supplementary Materials

The following supporting information can be downloaded at: https://www.mdpi.com/article/10.3390/ph19030512/s1, Table S1. ESI–MS/MS Parameters and analytical characteristics for the Analysis of Target Analytes by MRM Negative and Positive Ioni-zation Mode; Table S2. Calibration curves and sensitivity properties of the method; Table S3. Spearman rank correlation coefficients among antioxidant assays, enzyme inhibitory activities, phenolic constituents and the relative antioxidant capacity index (RACI).
